# The role of heavy metals and polychlorinated biphenyls (PCBs) in the oncogenesis of head and neck tumors and thyroid diseases: a pilot study

**DOI:** 10.1007/s10534-018-0091-9

**Published:** 2018-03-08

**Authors:** V. Petrosino, G. Motta, G. Tenore, M. Coletta, A. Guariglia, D. Testa

**Affiliations:** 1 ASL Salerno, Salerno, Italy; 2 ENT Clinic, University of Naples, via S. Pansini, 5, 80131 Naples, Italy; 3 Pharmaceutical Chemistry, University of Naples, Via Domenico Montesano, 49, 80131 Naples, Italy

**Keywords:** Heavy metals, PCB, Oncogenesis, Head and neck tumors

## Abstract

Previous literature has highlighted the mechanisms of molecular toxicity induced by substances such as arsenic, cadmium, chromium, nickel, lead, barium and PCBs. The research was carried out on 20 volunteers, all the patients gave their consent to the research: the aim of the study was to evaluate the presence of metals and PCBs in these different matrices (blood and hair), correlating the biochemical data to pathological conditions present, and also to the area in which patients resided. Various quantitative determinations were carried out on samples of blood and hair for 14 heavy metals and on blood samples for 12 PCBs. For the 11 patients the results indicated that blood levels for half of the 14 displayed heavy metals measured considerably higher compared to the reference values, whilst the levels measured in hair evidenced some positive values significantly higher than the maximum reference. Of the 12 PCBs assayed in blood some showed higher positive values compared to the maximum tabular reference (although there is no clear reference quantified in the WHO-2005 report). In the 9 healthy patients heavy metals in the blood were within the expected target range, with those showing positive results (≤ 3 out of 14 heavy metals for each patient) having values only slightly higher than the reference maximum. The levels of 14 heavy metals measured in hair were below thresholds, and levels for the 12 PCBs measured in blood showed negativity or positivity with values close to the minimum benchmarks. The analyses carried out on biological matrices have uncovered important and significant differences between healthy and unhealthy subjects, both qualitative and quantitative differences with respect to heavy metals and PCBs. All patients with head and neck cancer enlisted for the study had heavy metal and PCB blood levels at least twice the maximum reference level. The levels of heavy metals in hair were at least double the maximum reference. In contrast, all healthy volunteers enrolled showed no significant levels for either metals or PCBs.

## Background

Heavy metals and polychlorinated biphenyls (PCBs) can be found in many environmental settings, especially in areas where hazardous waste is willfully or negligently disposed of. These substances, characterized by toxicity and carcinogenicity, bioaccumulate and act as endocrine disruptors (Sukdolová et al. 2000); however, the relationship between the magnitude of exposure to these elements and the onset of neoplastic diseases is still a matter of investigation (Kim et al. 2015; Sa and Bz 2014). In addition, some of these elements require specific analytical methods for their determination, because they are active at picogram concentrations, and the maximum rates of their physiological absorption in humans are still unknown. It is also a challenge to understand how multiple chemical elements interact with each other, and with the human body (Kucharzewski et al. 2003; Langer et al. 2005). PCBs are molecules that were first synthesized at the beginning of the last century, so they do not exist in nature, but have been produced by industrial processes. PCBs are very stable compounds, poorly soluble in water, and highly lipophilic; they are derived from crude oil or coal tar, from which benzene is extracted and subsequently transformed into biphenyl, and have been used in many industrial productions (Sukdolová et al. 2000). They were used in the past in electrical transformers as oil insulation, as well as in electrical capacitors, sealants, paints, glues, printing inks, additives for pesticides, clearances for electrical conductors, photocopying papers, carbon papers, and in the production of many synthetic fibers (Kim et al. 2015; Sa and Bz 2014; Kucharzewski et al. 2003; Langer et al. 2005; Violante et al. 2000; Montes-Grajales et al. 2016; Carpenter 2006). They can also be produced by waste incineration, especially of PCBs containing oils. Even though many PCBs were banned after 1985, their presence in landfills and in many common everyday products is still a major source of pollution. PCB production seems to have been millions of tons, and they can be found almost everywhere, in marine sediments and rivers often as a result of wilful or negligent disposal of waste (Carpenter 2006). Most PCBs are introduced into the body by ingestion of contaminated food and water (Kim et al. 2015; Carpenter 2006). They have also the ability to bioaccumulate (Sa and Bz 2014; Carpenter 2006). These substances have been considered carcinogenic to humans by the International Agency for Research on Cancer (IARC) (Carpenter 2006; IARC 2012; WHO 2007). Some PCBs act on the arylhydrocarbon (AhR) receptor, but they also affect the immune system by stimulating the response of inflammatory mediators, and act as endocrine disruptors, as well as having genotoxic effects (Chung et al. 2015). Although there is no universally accepted definition of “heavy metals,” most of the metals with atomic number greater than 20 or whose density is greater than 5 g/cm^3^ are considered as heavy metals (Kim et al. 2015; Carpenter 2006).

Metals are present in the air, water, food, and are often dispersed in the atmosphere and soil as a result of industrial activities; some are essential—i.e., they are required by our body—but in high concentrations may become toxic (chromium, iron, copper, and zinc), while others do not play any specific roles in life processes (aluminum, nickel, arsenic, cadmium, mercury, and lead) (Kim et al. 2015; WHO 2007).

Heavy metals are byproducts of incinerators, combustion of gasoline or diesel fuel (cars, trucks, airplanes), smelters, paints, insecticides, and agriculture products such as disinfectants (Lauby-Secretan et al. 2013); they can be absorbed by inhalation, ingestion, or even skin contact, although to a lesser extent (Kucharzewski et al. 2003). Any of these metals, at high concentrations, can cause acute intoxication, and may affect multiple organs and systems (Kucharzewski et al. 2003). Several metals have been classified as definite or probable carcinogens by the IARC; arsenic, beryllium, cadmium, chromium, and nickel are carcinogenic (IARC 2012; Chung et al. 2015).

A number of studies in the literature have highlighted the molecular mechanisms of toxicity induced by specific metals, such as arsenic, cadmium, chromium, nickel, lead, and barium; according to the IARC, the damage occurs through oxidative stress, DNA modifications also due to epigenetic stress mechanisms, and as a result of their ability to act as endocrine disruptors (Yousaf et al. 2016; Chen et al. 2014; Sunderman 1981; Celetti et al. 2005; Staibano et al. 2007; Yao and Costa 2014; National Toxicology Program 2006).

The exposure of the population to chemicals in the environment and food is a major concern for healthcare institutions. We should begin to measure these substances in both sick and healthy individuals, and not only in the environment or in food, in order to detect their presence and the potential relationship with various diseases in the territory.

Head and neck cancers are a group of very common cancers worldwide, are the sixth most common malignancy in the world, and represent a significant problem especially in industrialized countries (Denaro et al. 2016). These cancers are commonly associated with alcohol consumption, tobacco use and abuse, and infection with human papilloma virus (HPV), especially with HPV16 (Blot et al. 1988; Hashibe et al. 2009). Excessive alcohol intake combined with smoking increases the risk of developing these tumors (Blot et al. 1988).

Exposure to chemical and physical agents (e.g., occupational exposure to wood dust), long-term exposure to second-hand smoke, improper oral hygiene, family history of cancer, and a diet low in vegetables are also risk factors for these types of cancers (Wozniak et al. 2016).

Approximately 25,000 new cases of head and neck cancers (including those of the thyroid) are diagnosed every year in Italy. In the majority of cases (over 90%), these cancers are squamous cell carcinomas developing from the epithelial tissue lining the mucosa of the district (AIOM 2015). There are also less common tumors that may originate from other tissues such as adenocarcinomas from the salivary glands, melanomas from melanin-producing cells, and lymphomas from lymphoid tissues (AIOM 2015).

## Study aim

This pilot study was carried out with the aim to determine the presence of heavy metals and PCBs in individuals with neoplastic diseases of the ENT/head and neck region, who were residents in geographic areas declared at risk, or possibly at risk of contamination—i.e., Naples and its province, Caserta and its province, Salerno and its province—taking into account the years of residence in these areas.

A total of 14 heavy metals were measured in the blood and in the hair (aluminum, antimony, arsenic, barium, cadmium, chromium, iron, lithium, mercury, nickel, lead, copper, strontium, and zinc), and a total of 12 PCBs (3,4,4′,5 *Tetrachlorobiphenyl,* 3,3′,4,4′ *Tetrachlorobiphenyl,* 2′,3,4,4′,5 *Pentachlorobiphenyl,* 2,3′,4,4′,5 *Pentachlorobiphenyl,* 2,3,4,4′,5 *Pentachlorobiphenyl,* 2,3,3′,4,4′ *Pentachlorobiphenyl,* 3,3′,4,4′,5 *Pentachlorobiphenyl,* 2,3′,4,4′,5,5′*Hexachlorobiphenyl,* 2,3,3′,4,4′,5 *Hexachlorobiphenyl,* 2,3,3′,4,4′,5′ *Hexachlorobiphenyl,* 3,3′,4,4′,5,5′ *Hexachlorobiphenyl,* 2,3,3′,4,4′,5,5′ *Heptachlorobiphenyl*) were measured in the blood.

The aim of this study was to assess the relationship and the presence of these metals in two different biological matrices (blood and hair), and the presence of PCBs, correlating the biochemical data with the pathological conditions as well as with the place of residence.

## Methods

The research was carried out on 20 volunteers, from whom blood and hair samples were taken (Table 1), and 9 were healthy individuals (Table 2), the present study was performed in accordance with the institutional review board guidelines, as well as the Helsinki Declaration of 1983, and it has been reviewed and approved by the Ethics Committee of the School of Medicine Second University of Naples, Italy (n. 2/15).

**Table 1:** Eleven patients with disease

ID	Sex	Disease	Province	Place of residence
1	M	Laryngeal cancer	NA	Casoria
2	F	Nasopharyngeal cancer	NA	Napoli
3	F	Laryngeal cancer	NA	Napoli
4	M	Non-Hodgkin’s lymphoma of the tonsil	CE	Frignano
5	M	Thyroid cancer	NA	Acerra
6	F	Thyroid cancer	NA	Casalnuovo
7	M	Thyroid cancer	SA	Cava de Tirreni
8	F	Thyroid cancer	NA	Aversa
9	F	Thyroid goiter	NA	Giugliano
10	F	Thyroid goiter	NA	Marano
11	F	Nodular thyroid disease	PZ	Bucaletto

**Table 2:** Nine healthy controls

Age	Province	Place of residence
52	SA	Cava de Tirreni
27	SA	Cava de Tirreni
9	PZ	Sant’Angelo Le Fratte
14	PZ	Sant’Angelo Le Fratte
47	PZ	Sant’Angelo Le Fratte
39	PZ	Brienza
39	PZ	Sant’Angelo Le Fratte
38	NA	Palma Campania
35	PZ	Sant’Angelo Le Fratte

In all 20 volunteers, after informed consent was obtained, a detailed medical history was taken with particular attention to medications used, place of usual residence, years of residence, and any major environmental concerns in the area. Quali–quantitative determinations of 14 heavy metals on blood and hair samples, and of 12 PCBs on blood samples were also performed. Blood and hair samples were taken during the patient’s stay in the hospital, where they underwent diagnostic and therapeutic work-up based on the disease they were suffering from, or at the Faculty of Pharmacy in Naples.

Blood and hair (0.5 g) samples underwent acid digestion with H_2_SO_4_ in a Ethos One microwave digester for 10 min at T = 200 °C, and Power = 1000 watts. The digested sample was added with 5 mL di HNO_3_ e 2 mL of H_2_O_2_ followed by mineralization in a microwave digestion system for 20 min at T = 200 °C, and Power = 1000 W.

Analyses were then performed using atomic absorption spectrophotometry technique with atomization in a graphite furnace, with the amounts of each element expressed in μg/100 g of sample. PCBs were determined, after partitioning with acetonitrile, and sulfur elimination, using purification-fractionation techniques by silica gel chromatography, and gas chromatography-mass spectrometry. All analyses on blood and hair samples were performed at the Department of Pharmacy of the University “Federico II” of Naples. All 11 individuals with tumors underwent a diagnostic protocol for cervicofacial tumors as recommended by guidelines, and were surgically treated based on the stage of the disease (Table 1).

## Results

**Fig. 1:**
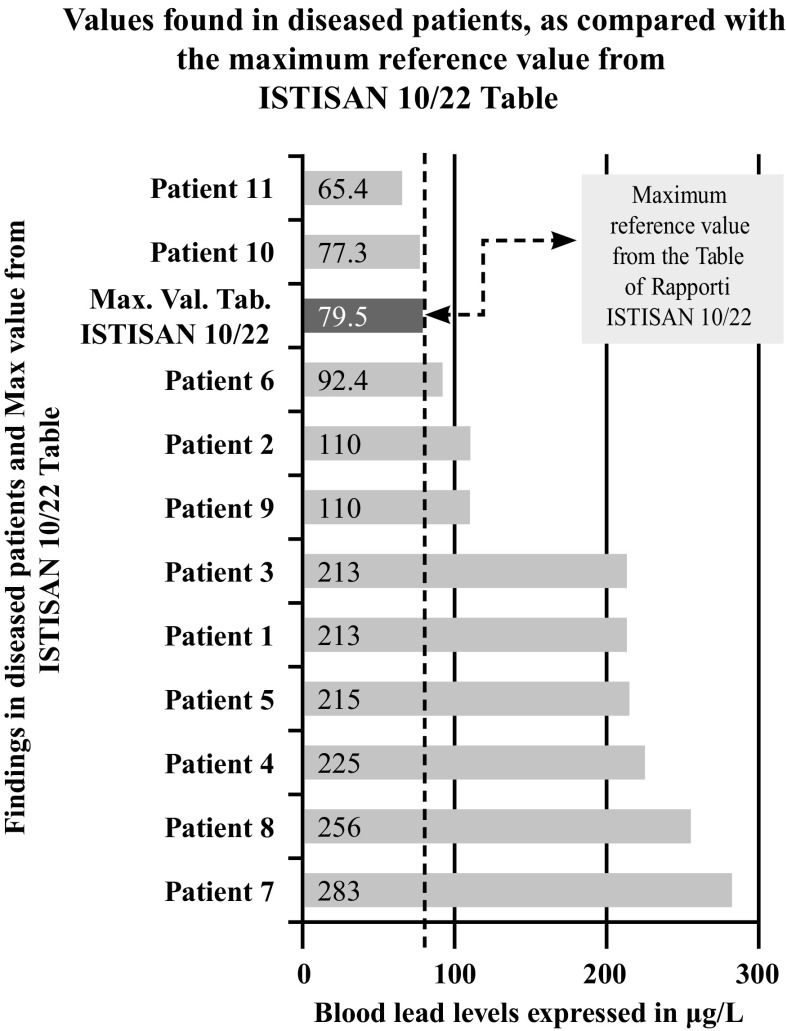
Blood Pb levels in the 11 diseased patients with regard to ISTISAN 10/22 Table (min 12.8; max 79.5)

The results of the analyses, expressed in µg/L in the blood and in µg/g in the hair for the 14 metals, and in pg/mL for PCBs, and evaluated according to the reference tables of the ISTISAN (Table 3) and WHO-2005 reports, were subsequently compared with the diseases each patient was suffering from, and to patient origin. The results showed that, in the 11 patients with tumors, the levels of the 14 heavy metals in the blood were considerably higher than the permitted levels, in half of them (e.g. lead) (Fig. 1), while the levels in the hair were significantly higher than the maximum reference values in some of them. With regard to the blood levels of the 12 PCBs, some of the patients showed positive values significantly higher than the maximum reference value (although there are no clearly established reference values according to the WHO-2005 reports).

**Table 3:** Blood references values from ISTISAN 10/22 table. Reproduced with permission from Rapporti ISTISAN 10/22

Metal	Min blood threshold value (μg/L)	Max blood threshold value (μg/L)
Aluminum	5.93	33.3
Antimony	0.07	0.94
Arsenic	0.4	11.9
Barium	0.5	2.4
Cadmium	0.25	1.97
Chromium	0.12	1.07
Copper	686	1157
Iron	453,519	646,491
Lead	12.8	79.5
Lithium	0.2	1.87
Mercury	1.7	9.9
Nickel	0.14	2.13
Selenium	85.4	277
Strontium	0.63	2.61
Zinc	5189	8337

**Fig. 2:**
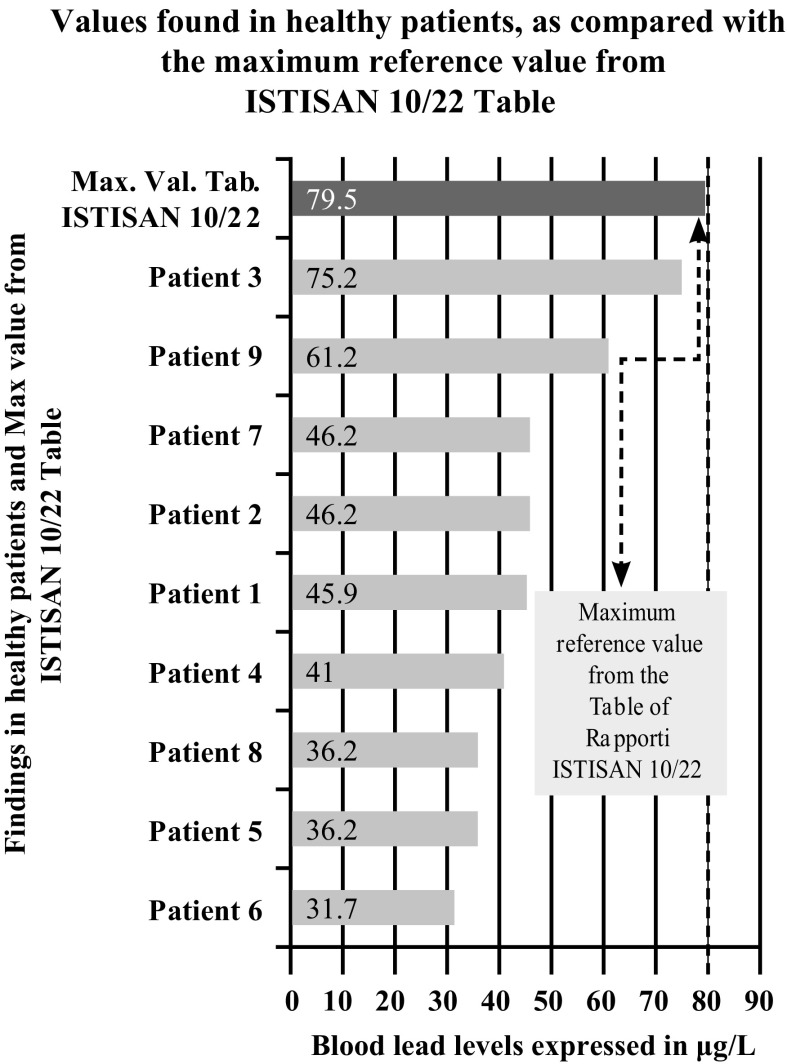
Comparison of the blood Pb levels in the 9 healthy patients

**Fig. 3:**
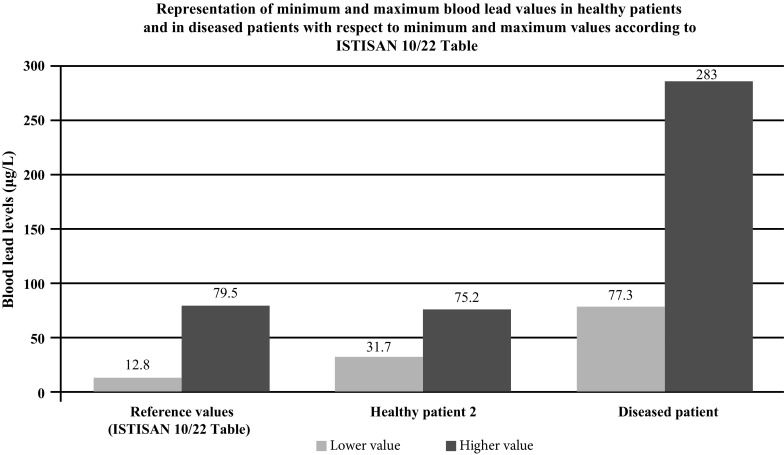
Minimum and maximum Pb values (according to ISTISAN Table) measured in the group of healthy patients and in the group of diseased patients

In the 9 healthy volunteers, concentrations of heavy metals in the blood were within the reference ranges; positive tested metals (≤ 3 of 14 heavy metals for each participant) were slightly higher than the maximum reference value (Figs. 2, 3). The levels of the 14 heavy metals in the hair were negative; the blood levels of the 12 PCBs were either negative or positive, with values close to the minimum reference value. The results of the tests performed on the 11 patients with tumors showed that the 2 patients with laryngeal carcinoma (a 57-year female patient with a G2–G3 squamous cell carcinoma of the vocal cords, and a 70-year male patient with a squamous cell carcinoma of the epiglottis) had similar elevated levels of the same heavy metals in the blood (Aluminum, Antimony, Arsenic, Cadmium, Chromium, Mercury, Nickel, Lead, Copper, Zinc), and of the same PCBs in the blood (2′3,4,4′,5-pentachlorobiphenyl [PeCB] [PCB-123]; 2,3′,4,4′,5-PeCB [PCB-118]; 2,3,4,4′,5-PeCB [PCB-114]); they had also similar elevated levels of Arsenic, Cadmium, Chromium and Lead in the hair. The patient suffering from non-Hodgkin’s lymphoma of the tonsil (mantle cell variety) had similar high levels in the blood as those found in the patients with laryngeal carcinoma (Aluminum, Antimony, Arsenic, Cadmium, Chromium, Mercury, Nickel, Lead, Copper), with the exception of zinc which was within the normal range, as well as high blood concentrations of the following PCBs: 2′,3,4,4′,5–2,3,4,4′5 and 2,3,3′,4,4′,5′-hexachlorobiphenyl (HxCB) (PCB-157). The levels of arsenic and chromium were the highest also in the hair. This patient also had the highest levels of aluminum, antimony, arsenic, cadmium, chromium, and nickel in the blood.

The 4 patients with thyroid carcinoma, both papillary and follicular types, had almost similar high levels of Aluminum, Cadmium, Mercury, Nickel, and Lead in the blood, as well as elevated blood levels of PCB-114; 2,3,4,4′,5.

Chromium levels were about three times higher in the hair, except in a case of papillary carcinoma where chromium values were close to the maximum permitted levels, and arsenic values were almost double. In 3 cases, we found both 2,3,4,4′,5- and 2,3′,4,4′,5-pentachlorobiphenyl in the blood.

All 3 patients with thyroid goiter had similarly high levels of Aluminum, Antimony, and Zinc in the blood; as for the PCBs, 2 patients had high blood levels of 2,3,3′,4,4′,5′-HxCB (PCB-156), and one patient had elevated blood levels of PCB-114. 2,3′,4,4′,5.

**Graph 1:**
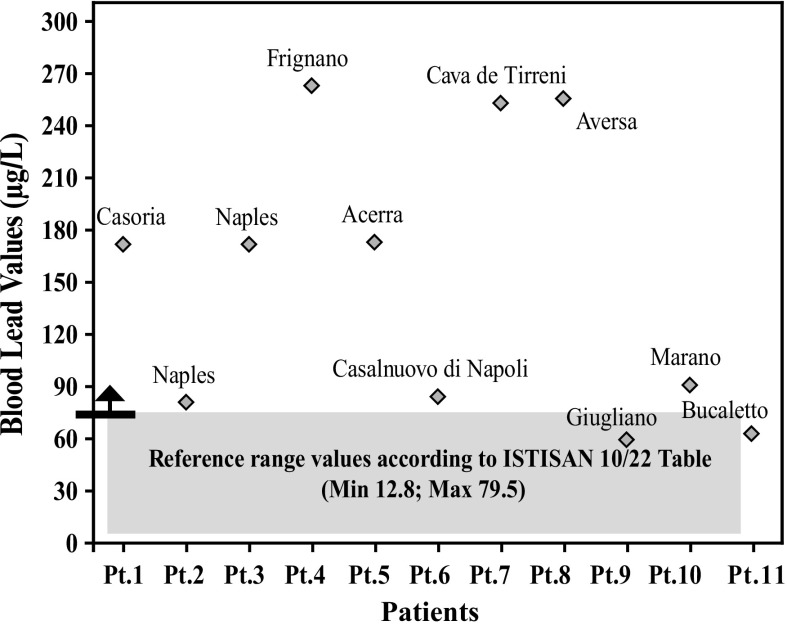
Blood concentration, expressed in μg/L, of lead according to the place of residence of diseased patients

**Graph 2:**
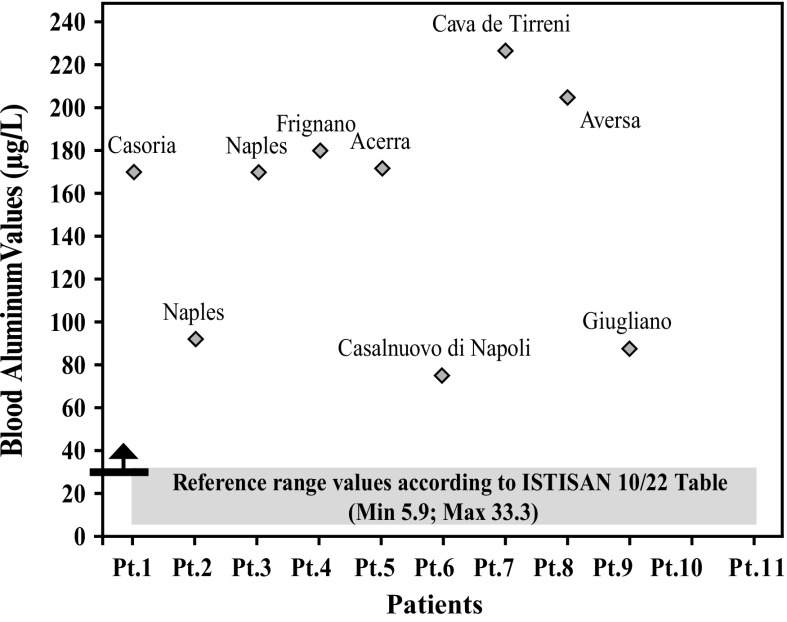
Blood concentration, expressed in μg/L, of aluminum according to the place of residence of diseased patients

**Graph 3:**
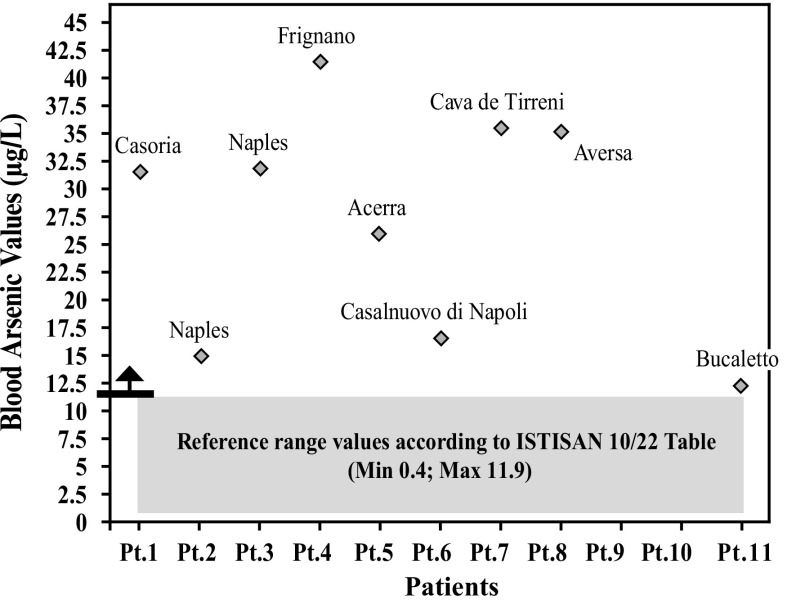
Blood concentration, expressed in μg/L, of arsenic according to the place of residence of diseased patients

**Graph 4:**
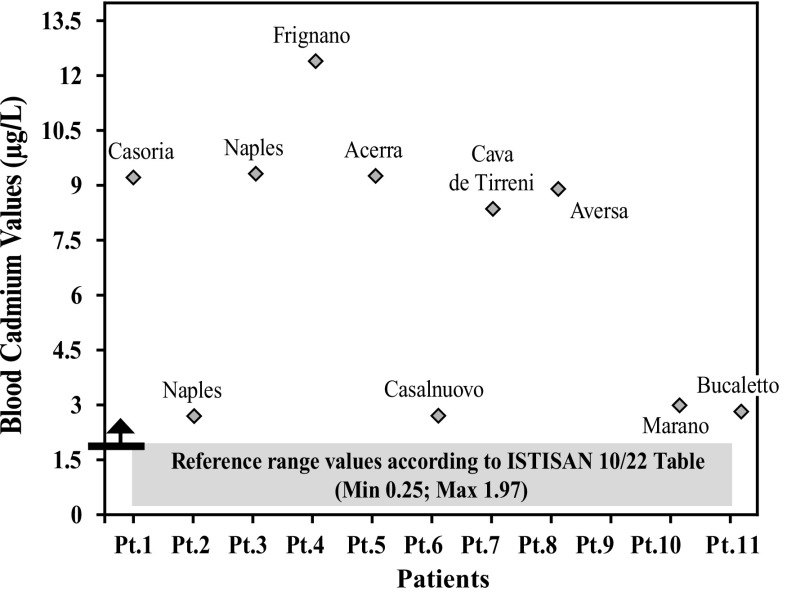
Blood concentration, expressed in μg/L, of cadmium according to the place of residence of diseased patients

**Graph 5:**
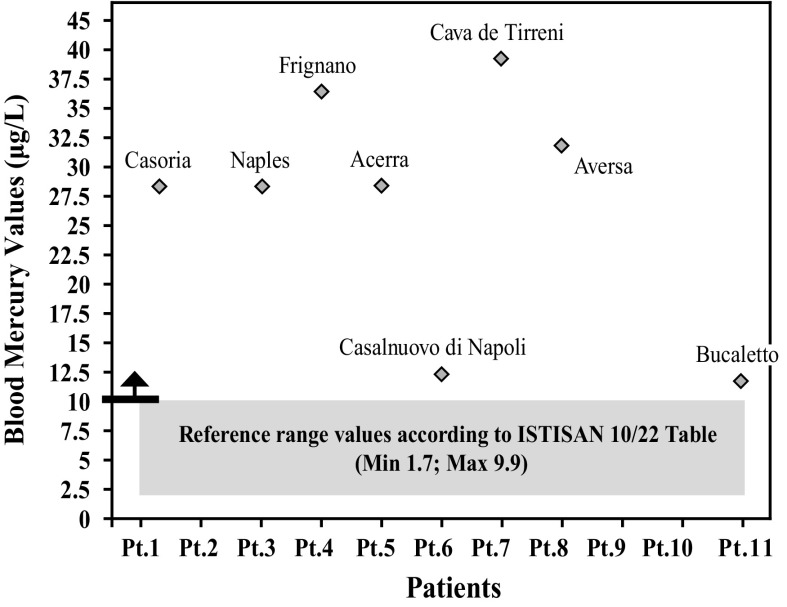
Blood concentration, expressed in μg/L, of mercury according to the place of residence of diseased patients

**Graph 6:**
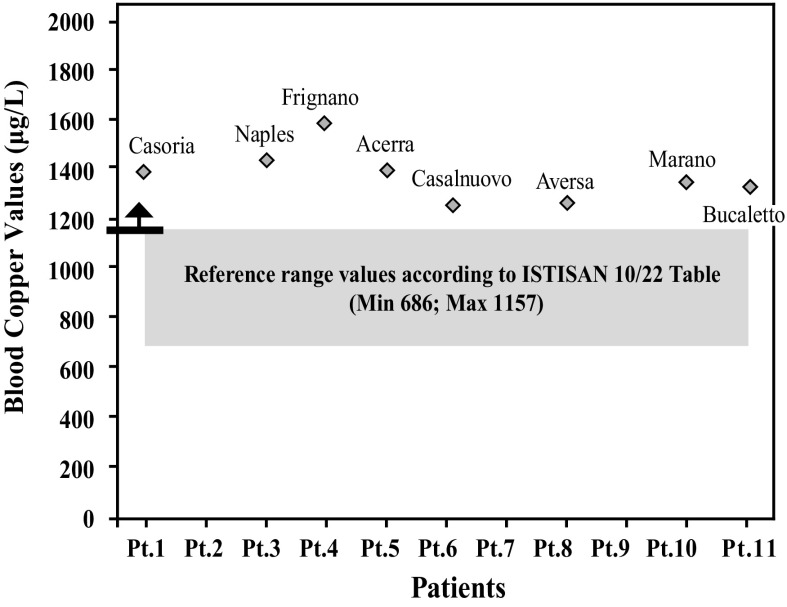
Blood concentration, expressed in μg/L, of copper according to the place of residence of diseased patients

**Graph 7:**
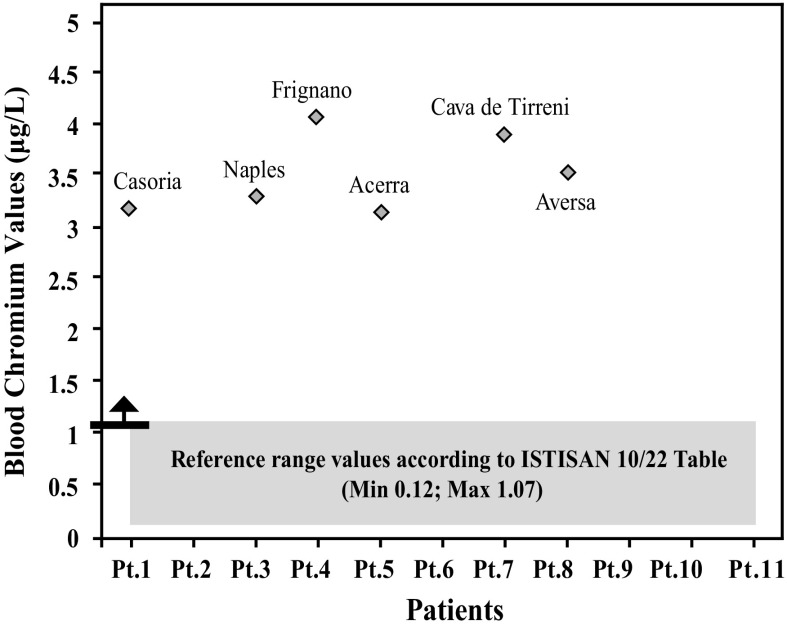
Blood concentration, expressed in μg/L, of chromium according to the place of residence of diseased patients

**Graph 8:**
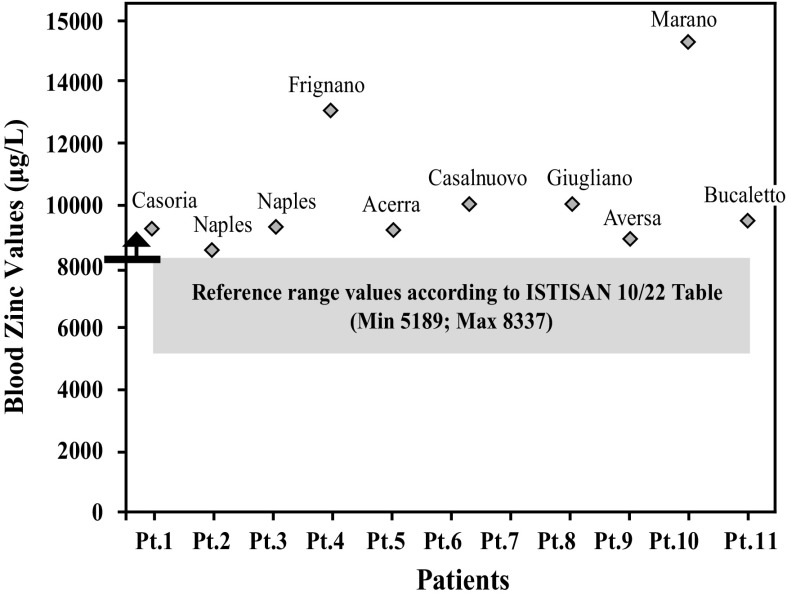
Blood concentration, expressed in μg/L, of zinc according to the place of residence of diseased patients

After further analysis of these findings, with patients di vided according to their place of residence, and compared with each other (Graphs 1, 2, 3, 4, 5, 6, 7, 8), we found that: in 4 patients from the area of Casoria (Na), Acerra (Na), Casalnuovo (Na), and Aversa (Na), test results showed commonly and significantly elevated levels of Aluminium, Cadmium, Mercury, zinc and lead measured in the blood; chromium was elevated in the blood in three patients from these towns, and was present in the hair of all four subjects; a patient from Giugliano (Na) showed high levels of Antimony, lead and zinc in the blood, and of Chromium and Arsenic in the hair; a patient from Marano (Na) showed high levels of Aluminum, Zinc, Cadmium, Antimony, and Chromium in the blood, and of Lead, cadmium in the hair; in 2 patients from Naples, higher values of Aluminum, Lead, Arsenic, Mercury, Antimonium, Cadmium, Nickel and Zinc in the blood, and of Chromium, Cadmium, Arsenic and Lead in the hair, were commonly reported; a patient living in Frignano (Ce) had the highest values of Aluminum, Cadmium, Arsenic, Mercury, and Lead in the blood, and of Chromium and Arsenicum in the hair; patients living in Cava de Tirreni (Sa) had higher values of Aluminum, Antimony, Arsenic, Cadmium, Chromium, Mercury, Nickel, and Lead in the blood, and of Arsenicum in the hair. The patient living in Buccaletto had elevated blood levels of aluminum, cadmium, nickel, and zinc, as well as elevated hair levels of chromium.

We did not find a clear correlation between tumor stage and the levels of metals in the blood and hair, or the levels of PCBs in the blood. Instead, we have shown above that the nasopharyngeal cancer and lymphoma of the tonsil were associated with the same heavy metals in the blood and with the same PCBs. The nasopharyngeal cancer had in the hair matrix the same metals that were present in the blood, and the lymphoma showed high levels of arsenic and chromium also in the hair matrix. Similarly, we have observed the same trend for thyroid tumors with the same metals in the blood (Aluminum, Cadmium, Mercury, Nickel, and Lead)

## Discussion

Normally, the matrices in which metals and PCBs are measured should be easily accessible matrices in order to obtain readily available material with a minimally invasive technique (Rapporti ISTISAN 10/22). We have used blood and hair matrices also to detect any possible differences. Hair matrix is often used because of its little or no invasiveness by many companies that advertise food supplements, or by private laboratories that perform “mineralograms,” which seems to have become a popular trend around the world in recent years.

The blood matrix detects short-, medium- and long-term exposures; the established minimum and maximum limits are relatively well standardized. The hair matrix should provide information especially on medium- and long-term exposures. Hair is considered somewhat as a waste bin, but is more susceptible to external contamination and changes such as hair age, use of shampoos, conditioners and dyes, so that false indications are possible, and minimum and maximum limits are less standardized; as a result, some determinations of heavy metals have not been well defined. Therefore, we do not believe that this matrix is very reliable. Moreover, we believe that, to obtain valid indications, multiple biological matrices should be carefully analysed.

Numerous studies have suggested the possibility of a causal relationship between onset of cancer and exposure to environmental carcinogens, particularly the correlation with the environmental presence of heavy metals and PCBs, as mechanistic determinants of oncogenesis (Capen 1994).

This study, which is part of a multidistrict research that takes into consideration different pathologies, considered only ENT patients with head and neck tumors, and was carried out on individuals living in recognized critical environmental areas.

The results of analytical tests performed on two biological matrices revealed important and significant differences, both qualitative and quantitative, between patients and healthy subjects in the concentrations of heavy metals and PCBs. To our knowledge, no similar study has been performed worldwide on two biological matrices simultaneously, and taking into account both the heavy metals and that particular group of 12 PCBs we measured. A group of researchers recently published a study that took into account the concentrations of 10 metals in the hair of patients with head and neck tumors. The researchers took into consideration lead, magnesium, iron, zinc, selenium, copper, manganese, calcium, and cobalt, and therefore not all the metals and all the established heavy metals (Wozniak et al. 2016). These researchers concluded that high levels of toxic metals in the blood may be evidence of current pathological processes (Wozniak et al. 2016). Exposure to some of these metals such as lead, cadmium, chromium, copper, and zinc can impair many functions of the body. Toxic doses of the above elements may lead to carcinogenesis, as confirmed by numerous studies (Patrick 2006; Gàl et al. 2008; Soudani et al. 2010; Templeton and Liu 2010; Hordyjewska et al. 2014). For example, lead is a highly toxic metal that can accumulate in the body and damage many organs and systems. It is considered a mutagenic element, among other reasons due to its lipid peroxidation enhancing effect (Afridi et al. 2011). Lead may also be found in high concentrations in the blood and urine of smokers (Afridi et al. 2011).

All patients with head and neck tumors enrolled in our pilot study had high blood levels of both heavy metals and PCBs, at least two times greater than the maximum reference value. The levels of heavy metals in hair were at least twice the maximum reference value; on the contrary, all enrolled healthy volunteers did not test significantly positive for metals or PCBs.

Patients coming from urbanized areas and living for several years close to areas considered at risk of wilful or negligent pollution. It would be appropriate to limit the exposure of the population to these substances by implementing environmental preventive measures, as well as the disposal of chemicals, and to initiate the remediation of areas declared at risk.

Many studies have shown that a greater consumption of fruit and vegetables reduces the risk of cancers developing in this body region (Li et al. 2012; Lucenteforte et al. 2009) have shown in a meta-analysis that fruit and vegetables are the most important and the most desirable diet ingredient in patients with mouth and laryngeal cancers.

However the real involvement of these substances and their intimate role in the oncogenesis of different tumors are increasingly being investigated by the international scientific community. Nevertheless, the preliminary finding of this pilot study clearly demonstrate the presence of high concentrations of heavy metals and PCBs in patients with head and neck tumors.

## Conclusions

The analyses carried out on biological matrices have uncovered important and significant differences between healthy and unhealthy subjects, both qualitative and quantitative differences with respect to heavy metals and PCBs.

All patients with head and neck cancer enlisted for the study had heavy metal and PCB blood levels at least twice the maximum reference level. The levels of heavy metals in hair were at least double the maximum reference. In contrast, all healthy volunteers enrolled showed no significant levels for either metals or PCBs.

The actual correlation of these substances to a definitive role in oncogenesis of cancer pathologies is now being investigated by the international scientific community, our findings demonstrate a high concentration of heavy metals and PCBs in patients with head and neck cancer.

### Availability of data and material

All demographic data, raw data, and results of the laboratory tests performed in this study are available from and in the possession of the corresponding author.

## Abbreviations

PCB: Polychlorinated biphenyls
WHO: World Health Organization
IARC: International Agency for Research on Cancer
AhR: Arylhydrocarbon
ENT: Ear, nose, and throat
ISTISAN: Istituto Superiore di Sanità
PeCB: Pentachlorobiphenyl
HxCB: Hexachlorobiphenyl

## References

[CR1] Afridi H, Brabazon D, Kazi T (2011). Evaluation of essential trace and toxic elements in scalp hair samples of smokers and alcohol user hypertensive patients. Biol Trace Elem Res.

[CR2] AIOM (2015) AIOM Linee Guida—Tumori della testa e del collo—Edizione. http://www.aiom.it/

[CR4] Blot WJ, McLaughlin JK, Winn DM (1988). Smoking and drinking in relation to oral and pharyngeal cancer. Cancer Res.

[CR5] Capen CC (1994). Mechanisms of chemical injury of thyroid gland. Prog Clin Biol Res.

[CR6] Carpenter DO (2006). Polychlorinated biphenyls (PCBs): routes of exposure and effects on human health. Rev Environ Health.

[CR7] Celetti D, Testa S Staibano (2005). Overexpression of the cytokine osteopontin identifies aggressive laryngeal squamous cell carcinomas and enhances carcinoma cell proliferation and invasiveness. Clin Cancer Res.

[CR8] Chen YY, Zhu JY, Chan KM (2014). Effects of cadmium on cell proliferation, apoptosis, and proto-oncogene expression in zebrafish liver cells. Aqua Toxicol.

[CR9] Chung HK, Nam JS, Ahn CW (2015). Some elements in thyroid tissue are associated with more advanced stage of thyroid cancer in Korean women. Biol Trace Elem Res.

[CR10] Denaro N, Merlano MC, Russi EG (2016). Follow-up in head and neck cancer: do more does it mean do better? A systematic review and our proposal based on our experience. Clin Exp Otorhinolaryngol.

[CR11] Gàl J, Hursthouse A, Tatner P (2008). Cobalt and secondary poisoning in the terrestrial food chain: data review and research gaps to support risk assessment. Environ Int.

[CR12] Hashibe M, Brennan P, Chuang SC (2009). Interaction between tobacco and alcohol use and the risk of head and neck cancer: pooled analysis in the International Head and Neck Cancer Epidemiology Consortium. Cancer Epidemiol Biomarkers Prev.

[CR13] Hordyjewska A, Popiołek Ł, Kocot J (2014). The many ‘‘faces’’ of copper in medicine and treatment. Biometals.

[CR14] IARC (2012). IARC monographs on the evaluation of carcinogenic risk to human.

[CR15] Kim HS, Kim YJ, Seo YR (2015). An overview of carcinogenic heavy metal: molecular toxicity mechanism and prevention. J Cancer Prev.

[CR16] Kucharzewski M, Braziewicz J, Majewska U (2003). Copper, zinc, and selenium in whole blood and thyroid tissue of people with various thyroid diseases. Biol Trace Elem Res.

[CR17] Langer P, Kocan A, Tajtakova M, Petrik I (2005). Human thyroid in the population exposed to high environmental pollution by organochlorinated pollutants for several decades. Endocr Regul.

[CR18] Lauby-Secretan B, Loomis D, Grosse Y (2013). Carcinogenicity of polychlorinated biphenyls and polybrominated biphenyls. Lancet Oncol.

[CR19] Li Q, Chuang S, Eluf-Neto J, Menezes A, Matos E (2012). Vitamin or mineral supplement intake and the risk of head and neck cancer: pooled analysis in the INHANCE consortium. Int J Cancer.

[CR20] Lucenteforte E, Garavello W, Bosetti C (2009). Dietary factors and oral and pharyngeal cancer risk. Oral Oncol.

[CR21] Montes-Grajales D, Bernardes GJ, Olivero-Verbel J (2016). Urban endocrine disruptors targeting breast cancer proteins. Chem Res Toxicol.

[CR22] National Toxicology Program (2006). Toxicology and carcinogenesis studies of a binary mixture of 3,3′,4,4′,5-pentachlorobiphenyl (PCB 126) (Cas No. 57465-28-8) and 2,2′,4,4′,5,5′-hexachlorobiphenyl (PCB 153) (CAS No. 35065-27-1) in female Harlan Sprague-Dawley rats (gavage studies). Tech Rep Ser.

[CR23] Patrick L (2006). Lead toxicity, a review of the literature. Part 1: exposure, evaluation, and treatment. Altern Med Rev.

[CR24] Sa Jancic, Bz Stosic (2014). Cadmium effects on the thyroid gland. Vitam Horm.

[CR25] Soudani N, Sefi M, Ben Amara I (2010). Protective effects of selenium (Se) on chromium (VI) induced nephrotoxicity in adult rats. Ecotoxicol Environ Saf.

[CR26] Staibano S, Merolla F, Testa D, Iovine M (2007). Opn/Cd44v6 overexpression in laryngeal dysplasia and correlation with clinical outcome. Br J Cancer.

[CR27] Sukdolová V, Negoita S, Hubicki L (2000). The assessment of risk to acquired hypothyroidism from exposure to PCBs: a study among Akwesasne Mohawk women. Cent Eur J Public Health.

[CR28] Sunderman FW (1981). Recent research on nickel carcinogenesis. Environ Health Perspect.

[CR29] Templeton D, Liu Y (2010). Multiple roles of cadmium in cell death and survival. Chem Biol Interact.

[CR30] Violante N, Senofonte O, Marsili G (2000). I capelli umani come marcatore di inquinamento da elementi chimici emessi da una centrale termoelettrica. Istit Super Sanita Microchem J.

[CR31] WHO (2007). Health risks of heavy metals from long-range transboundary air pollution.

[CR32] Wozniak A, Napierala M, Golasik M (2016). Metal concentrations in hair of patients with various head and neck cancers as a diagnostic aid. Biometals.

[CR33] Rapporti ISTISAN 10/22 http://www.iss.it/binary/publ/cont/10ventidueWEB.pdf

[CR34] Yao Y, Costa M (2014). Toxicogenomic effect of nickel and beyond. Arch Toxicol.

[CR35] Yousaf B, Amina Liu G, Wang R (2016). The importance of evaluating metal exposure and predicting human health risks in urban-periurban environments influenced by emerging industry. Chemosphere.

